# Are Tai Chi and Qigong effective in the treatment of traumatic brain injury? A systematic review

**DOI:** 10.1186/s12906-024-04350-3

**Published:** 2024-02-06

**Authors:** Nicole Alexandra Laskosky, Patricia Huston, Wai Ching Lam, Charlotte Anderson, Linda L. D. Zhong

**Affiliations:** 1 Department of Family Medicine, Faculty of Medicine, University of Ottawa, Ottawa, Canada; 2 Institut du Savoir Montfort (Research), University of Ottawa, Ottawa, Canada; 3 School of Chinese Medicine, Hong Kong Baptist University, kowloon tong, Hong Kong, SAR, China; 4 School of Biological Sciences, Nanyang Technological University, Singapore, Singapore; 5 Faculty of Medicine, University of Toronto, Toronto, Canada

**Keywords:** Traumatic brain injury, Concussion, Tai chi, Qigong, Systematic review

## Abstract

**Background:**

Traumatic brain injury (TBI) adversely affects both young and old and is a growing public health concern. The common functional, psychological, and cognitive changes associated with TBI and recent trends in its management, such as recommending sub-threshold aerobic activity, and multi-modal treatment strategies including vestibular rehabilitation, suggest that Tai Chi/Qigong could be beneficial for TBI. Tai Chi and Qigong are aerobic mind-body practices with known benefits for maintaining health and mitigating chronic disease. To date, no systematic review has been published assessing the safety and effectiveness of Tai Chi/Qigong for traumatic injury.

**Methods:**

The following databases were searched: MEDLINE, CINAHL Cochrane Library, Embase, China National Knowledge Infrastructure Database, Wanfang Database, Chinese Scientific Journal Database, and Chinese Biomedical Literature Database. All people with mild, moderate, or severe TBI who were inpatients or outpatients were included. All Types of Tai Chi and Qigong, and all comparators, were included. All measured outcomes were included. A priori, we chose “return to usual activities” as the primary outcome measure as it was patient-oriented. Cochrane-based risk of bias assessments were conducted on all included trials. Quality of evidence was assessed using the grading of recommendation, assessment, development, and evaluation (GRADE) system.

**Results:**

Five trials were assessed; three randomized controlled trials (RCTs) and two non-RCTs; only two trials were conducted in the last 5 years. No trial measured “return to normal activities” or vestibular status as an outcome. Four trials - two RCTs and two non-RCTS - all found Tai Chi improved functional, psychological and/or cognitive outcomes. One RCT had a low risk of bias and a high level of certainty; one had some concerns. One non-RCTs had a moderate risk of bias and the other a serious risk of bias. The one Qigong RCT found improved psychological outcomes. It had a low risk of bias and a moderate level of certainty. Only one trial reported on adverse events and found that none were experienced by either the exercise or control group.

**Conclusion:**

Based on the consistent finding of benefit in the four Tai Chi trials, including one RCT that had a high level of certainty, there is a sufficient signal to merit conducting a large, high quality multi-centre trial on Tai Chi for TBI and test it against current trends in TBI management. Based on the one RCT on TBI and Qigong, an additional confirmatory RCT is indicated. Further research is indicated that reflects current management strategies and includes adverse event documentation in both the intervention and control groups. However, these findings suggest that, in addition to Tai Chi’s known health promotion and chronic disease mitigation benefits, its use for the treatment of injury, such as TBI, is potentially a new frontier.

**Systematic review registration:**

PROSPERO [CRD42022364385].

**Supplementary Information:**

The online version contains supplementary material available at 10.1186/s12906-024-04350-3.

## Introduction

Traumatic brain injury (TBI) is a disruption of normal neurological function resulting from a physical assault on the head, neck, or elsewhere on the body, leading to physical, cognitive, and emotional effects [1–4]. TBI contributes to a substantial proportion of the global injury burden, ranking as one of the top 10 neurological causes for high rates of disability adjusted life-years, and incidence rates continue to climb worldwide [5]. Falls and road injuries are the leading causes of new cases globally, particularly in the middle-aged and elderly; however, in North America, sport-related injuries in children and adolescents account for a significant proportion of new TBI cases [3, 4, 6, 7]. Since 2005, TBI incidence rates in Canada have more than doubled and these rates are expected to increase, suggesting a significant burden on the Canadian economy and healthcare system [3, 4, 6, 7].

The presentation of TBI is typically non-specific and widely varies. Somatic symptoms often include headache, fatigue, and signs of vestibular dysfunction, including dizziness, vertigo, and loss of balance [1, 2, 8]. Cognitive impairment is common, resulting in decreased concentration and impaired academic and job performance [2, 9]. When cognitive function decreases, this is often associated with anxiety and depression.

Recent research has furthered our understanding of the pathophysiology of TBI. Computer tomography (CT) has found that about 16% of patients with mild TBI have macrostructural intracranial injuries, including cerebral contusions and subdural hematomas [10]. When blood vessels are damaged, surrounding brain cells die and release damage associated molecular patterns (DAMPs), which promote the release of pro-inflammatory cytokines [11]. While an acute inflammatory response is part of the normal repair cycle to remove dead cells and debris, an excessive release of inflammatory cytokines can result in prolonged inflammation and brain damage [12]. Furthermore, this can manifest as neuropsychological sequala, including fatigue, headache, anxiety, and depression [13].

Standard care for TBI is initial physical and cognitive rest, education, and a gradual return to play, school, work, or usual activities [14, 15]. Despite advancing research, there is no evidence in favor of pharmacological treatment, and the current evidence suggests that anti-inflammatory medications should not be given for the treatment of TBI [16]. Although it was once thought that only 20% of the TBI population had persistent symptoms at 1-year post-injury [17], the recent TRACK-TBI study in the United States found that less than 50% of patients with mild TBI reported full return to pre-injury levels of functioning 1 year post-injury [18]. There is emerging evidence that TBI is associated with an increased risk of subsequent dementia and stroke and has led to recommendations to conduct more research, assess new treatments [19–21], and develop healthcare models that integrate medical and community services to support this patient population [2].

There have been several recent advances in the treatment of TBI. Based on several systematic reviews, it is now a best practice to include early sub-threshold aerobic activity for sport-related TBI [22, 23], and this has been assessed in the management of TBI from other causes [24]. In those with persistent symptoms, there is some evidence that individually tailored, multi-modal care (which includes supportive psychotherapy, cognitive rehabilitation, and cervical and vestibular rehabilitation) accelerates the return to normal activities, [15, 24]. Research has shown that vestibular rehabilitation can be particularly beneficial for patients with persistent vestibular symptoms of TBI, including dizziness, vertigo, and balance dysfunction [25–27].

Tai Chi (taijiquan or Tai Chi Chuan) and Qigong are aerobic mind-body practices based on common traditional Chinese medicine principles and as such, they are often studied together. Both Tai Chi and Qigong are known to increase fitness and well-being. Tai Chi is also considered to be a martial art [28–30]. There is good evidence that Tai Chi mitigates the symptoms of a number of chronic diseases [31] and some evidence that Qigong may as well [32].

Mind-body practices, such as meditation, yoga Tai Chi and Qigong, are typically practiced in community settings and have become increasingly common health-promoting activities. In the United States, one in four adults with neuropsychological symptoms report using mind-body practices, and often do not discuss this with their healthcare provider [33]. In Europe, similar findings have been reported for other health issues [34]. There is early evidence that mind-body practices can be helpful for people with TBI [35–37] and this may be due in part from reducing inflammation [38–40].

There is evidence that Tai Chi is effective for a number of the common symptoms associated with TBI, such as decreased function caused by impaired balance [41–44], psychological factors, such as anxiety and depression [45–47], cognitive impairment [48–51], and vestibular dysfunction [52, 53]. While less robust, there is good evidence that Qigong also has beneficial effects on balance [54, 55], psychological symptoms [47, 56], cognitive impairment [50, 51, 57] and there is preliminary evidence that Qigong may improve vestibular function [55].

There is also a growing body of evidence that indicates Tai Chi has beneficial effects on the brain. For example, since 2020, there have been at least seven systematic reviews showing Tai Chi improves mild cognitive impairment [48, 58–62]. Mechanistic studies have been conducted to try to explain this. A 2018 systematic review identified Tai Chi increases both brain connectivity and grey matter volume [63]. Since that time, multiple studies have confirmed these findings in young [64–66], middle-aged [67, 68], and older adults [69, 70]. There is preliminary evidence that Qigong may have similar effects [71, 72].

A large systematic review of Tai Chi trials have shown that it is safe with few adverse effects [73]; a systematic review to document and assess the safety of Qigong is planned [74]. Both can provide health benefits for both young and older adults at any fitness level [29, 31, 75–81].

To date, no systematic review has been published on Tai Chi/Qigong for TBI. Systematic reviews are indicated after the first few studies of a novel treatment to assess the accumulating evidence base, identify whether there is overall evidence of effectiveness and where uncertainties remain, and make recommendations for future studies. With increased emphasis on patient-centered care, the call to develop healthcare models that integrate medical and community services to support patients affected by TBI, and the evidence that Tai Chi/Qigong mitigates many of the symptoms of TBI, this novel treatment merits study. Therefore, the objective of this study was to conduct a systematic review to assess the safety and effectiveness of Tai Chi and Qigong for the treatment of traumatic brain injury.

## Methods

### Study registration

The systematic review protocol was registered on PROSPERO as “Are Tai Chi and Qigong Effective in the Treatment of Traumatic Brain Injury? A Systematic Review” (registration number: CRD42022364385). The systematic review was designed to be in accordance with the Preferred Reporting Items for Systematic Reviews and Meta-Analyses (PRISMA) guidelines [82]. As per best practices identified in these guidelines, the systematic review protocol was published [83].

### Inclusion criteria

We included all clinical trials on TBI that studied Tai Chi or Qigong alone or as a component of an intervention and had a comparison group, such as usual care, another exercise or non-exercise intervention, or no intervention (e.g., on a waiting list).

All participants in such studies were included regardless of age, sex, nationality, or whether they were inpatients or outpatients. All types of Tai Chi/ Qigong and lengths of intervention were included (Table 1).

**Table 1:** Inclusion criteria

**Design**	
Randomized and non-randomized clinical trials	
**Setting**	
Inpatient, outpatient, community-based	
**Participants**	
Individuals diagnosed with a concussion or traumatic brain injury (mild, moderate, or severe) All ages, sexes, races	
**Intervention**	
Tai Chi or Qigong as the primary intervention with or without another adjuvant therapy (e.g., rehabilitation or cognitive training) All styles and durations of study	
**Comparison**	
Any comparison group, such as: • Waiting list • Standard care • Any active control (such as physiotherapy, walking) • Any passive control (such as health education)	

### Outcome measures

As per recent trends to have patient-oriented outcomes [84], we identified the primary outcomes as return to school, sports, work, or usual activities. Secondary outcomes included any outcome measure, such as improvement in the common symptoms of TBI, quality of life, exacerbations, or the type and frequency of adverse events.

### Search strategy

We searched the following electronic databases: MEDLINE, CINAHL, Cochrane Library, Embase, China National Knowledge Infrastructure Database, Wanfang Database, Chinese Scientific Journal Database, and Chinese Biomedical Literature Database. To identify additional studies, ClinicalTrials.gov was searched for any new or planned trials and studies that may be in the grey literatures were searched in OpenGrey.eu. The last search was performed on January 25, 2023. Search terms included Tai Ji, T’ai Chi, Tai Chi, Taji, Chi Kung, Qigong, craniocerebral trauma, head or cranial trauma or injury, commotio cerebri, concussion, TBI, mild TBI, and related terms. Table 2 shows the search strategy for MEDLINE. The complete Chinese and English search strategy are given in Appendix 1. The reference lists of all identified studies and related systematic reviews were examined to identify additional studies.

**Table 2:** MEDLINE search strategy

1	exp Craniocerebral Trauma/
2	((head or crani* or capitis or brain* or forebrain* or skull* or hemisphere or intracran* or orbit* or cerebr*) adj2 (injur* or trauma* or lesion* or damage* or wound* or destruction* or oedema* or edema* or fracture* or contusio* or pressur*)).ti,ab,kf.
3	(mtbi or tbis or tbi).ti,ab,kf.
4	concuss*.ti,ab,kf.
5	commotio*.ti,ab,kf.
6	or/1–5
7	Tai Ji/ or Qigong/
8	(t'ai chi* or tai chi* or tai ji* or taiji* or qi gong* or qigong* or chi kung* or chikung*).ti,ab,kf.
9	or/7–8
10	6 and 9

### Data collection and analysis

All eligible English and Chinese studies identified during the database search were imported into Covidence, a production platform for systematic review article selection [85], and screened for duplicate documents. Three authors (NL, PH, WL) independently screened articles against the eligibility criteria based on title and abstract. Full-text articles for all eligible studies were independently assessed by all three authors against the inclusion criteria. The authors were blinded to each other’s decisions. Disagreements were resolved by discussion amongst the three main authors until an agreement was reached. The two other authors, CA and LZ, were available for consultation, if needed, to reach consensus.

Data were collected using standard excel data forms. Target population, diagnostic criteria, sample size, patient demographics, including age and sex, time from TBI to initiation of study, intervention program structure and details, control group details, all outcome measures, and follow up period were extracted. Two authors (PH, WL) independently extracted data and two other authors independently checked the extracted data (NL, CA). Study investigators were contacted for unreported or unclear data, such as intervention and control program structure. Data were reviewed and disagreements were resolved by discussion among all authors.

The Cochrane risk of bias tool (RoB 2.0) was used to assess randomized controlled trials (RCTs) and the risk of bias in non-randomized studies-of interventions (ROBINS-I) was used to assess observational studies [86, 87]. The grading of recommendation, assessment, development, and evaluation (GRADE) assessment was then conducted to evaluate the included studies [88]. The quality of evidence [89] was determined as very low, low, moderate, or high based on the GRADE considerations such as risk of bias, imprecision [90], inconsistency [91], indirectness [92], and publication bias [93]. Any disagreements were resolved by discussion amongst all authors.

## Results

### Search results

**Fig. 1:**
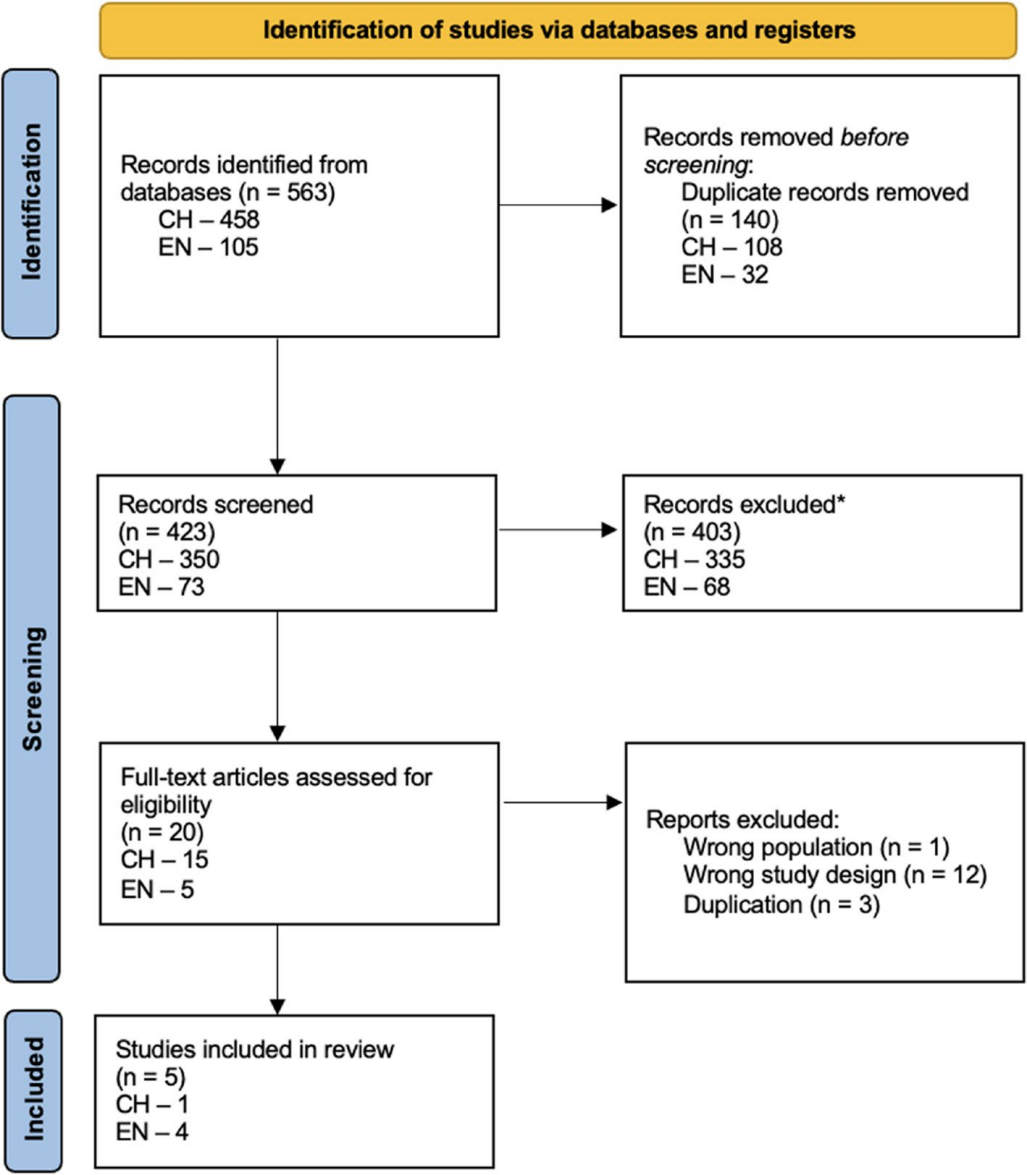
PRISMA diagram for Chinese and English databases study selection process. Note: CH, Chinese; EN, English *Reasons for exclusion from screening included: Case reports, retrospective cohort studies, case-control studies, systematic reviews

Among both the Chinese and English databases, a total of 563 studies were identified and after duplications were removed, 423 articles were screened. As per the PRISMA diagram, after screening the titles and abstracts, and full-text articles when indicated, five studies were included in the review (Fig. 1).

When references of six relevant systematic reviews were checked, no additional studies were identified. These reviews either included Tai Chi with other interventions, such as yoga or other community-based activities [37, 94–97], or assessed Tai Chi for neurologic disorders, including TBI [36]. A further study search via ClinicalTrials.gov did not identify any new or planned clinical trials on this topic, and a grey literature search on OpenGrey.eu did not yield any additional studies.

### Characteristics of included trials

In total, three RCTs and two non-randomized controlled trials (non-RCTs) met all the inclusion criteria. Four studies were published in English, one in Chinese. Each trial was conducted in a different country: United Kingdom [98], Poland [99], Taiwan [100], Mainland China [101], and New Zealand [102]. The oldest study was published in 2006 [102] and the most recent study in 2019 [100]. The sample size ranged from 18 to 98 for a total of 272 participants across all trials. Participant age ranged from 20 to 65 years and older. In four of the five trials, the causes of TBI were identified [98, 99, 101, 102]; most participants had been involved in a motor vehicle accident.

Three studies included participants with mild, moderate, or severe TBI [98, 100, 102], one trial included only those with severe TBI who had recently come out of a coma [99], and one trial noted patients who had TBI, but did not report on the level of severity [101]. One study identified that the diagnosis of TBI had been made by x-ray and cranial CT scan [101]. In the other studies, participants were included based on a previous diagnosis, such as the diagnosis noted on the discharge summary from hospital [98–100, 102].

There were different types of interventions, teacher qualifications, program intensities, and duration. Four studies assessed a Tai Chi-based intervention, and each was different: a 5-form Chen style [102], an 8-form Yang style [100], a 24-form style [101], and one did not identify the style of Tai Chi employed [99]. One study included an unspecified type of Qigong [98]. Three studies described qualifications of the instructors, such as a fully qualified Tai Chi instructor [102], instructor having at least 10 years’ experience [100], and an independent instructor [98]. One study noted a nurse demonstrated the Tai Chi moves [101], and in one study the instructor was not described [99]. The intensity of the intervention ranged from once a week to five times a week and the duration of the intervention ranged from 6 weeks to 6 months.

Three trials had no post-intervention follow-up [98, 99, 101], one trial had a follow-up at 3 weeks [102], and another at 6 months [100]. The Qigong group was compared to a group that participated in social and leisure activities [98]. Two Tai Chi studies were add-on trials where all participants received usual rehabilitative care [99, 101]. One Tai Chi trial had two control groups, usual care, and computerized cognitive training [100], and for one trial the control group was participants waiting to join a Tai Chi group [102].

There was significant diversity in the outcome measures between all 5 trials. None of the trials specifically measured return to school, sports, work, or usual activities. Moreover, none of the trials measured multi-modal treatment strategies including vestibular rehabilitation or assessed inflammatory markers. Four of the five trials measured participants’ psychological state: two by the Medical Outcomes Scale Short Form 36 (SF-36) [101, 102], and one each by the General Health Questionnaire [98], Rosenberg Self-Esteem Scale (RSES) [102], the Visual Analogue Mood Scale (VAMS) [102], and the Centre for Epidemiological Depression Scale [100]. Three studies measured physical fitness either through the Fugl-Meyer Assessment (FMA) [101], the Physical Self-Description Questionnaire [98], or by hand grip strength, sit-stand, and balance [100]. Two trials assessed activities of daily living (ADLs), through the Standard Self-Care Scale [99] or the Barthel Index [101]. One study, that only included subjects 55 years and older, measured cognitive status including the Mattis Dementia Rating Scale (MDRS), Mini-mental state examination (MMSE), modified Telephone Interview of Cognitive Status (TICS-M), and Trail Making Test (TMT) [100]. A summary of trial characteristics is noted in Table 3.

**Table 3:** Characteristics of clinical trials on Tai Chi and Qigong for traumatic brain injury

Study	Setting/Language	Type of study and participants	Intervention and Comparator	Outcomes
Gemmell 2006 [102]	Tai Chi school in New Zealand (English)	Single centre RCT (*n* = 18) Participants were 40–60 yrs. old, on av. 8.7 yrs. post mild/mod/severe TBI, from MVA and other causes (assumed ambulatory)	Fully qualified instructors taught the 5-Form Chen style Tai Chi for 45 min twice/wk. × 6 wks with 3 wk. follow-up Control: Wait list (to join Tai Chi grp)	Function: MOS-SF 36 Psychological: RSES, VAMS Adverse Events: Not reported Drop-outs: Not reported
Blake 2009 [98]	Community Centre in the UK (English)	Single centre RCT (*n* = 20) Participants were 20–63 yrs. old, 2–40 yrs. post TBI, 70% from MVAs, and 70% ambulatory	Independent instructor taught Qigong for one hour once/wk. for 8 wks. Control: Social/leisure activities	Psychological: General health, self-esteem, flexibility, coordination, Physical self description Q, Social Support for Exercise Habits Scale. Adverse Events: No adverse events reported in either group Drop outs: 1 (in control group)
Manko 2013 [99]	Hospital in Poland (Polish)	Single centre comparative trial (*n* = 40) Participants were on av. 33.6 yrs. old in Tai Chi grp (26 yrs. control grp) with severe TBI recently in coma. Time from TBI not reported (assumed non-ambulatory at first)	Add-on design: both grps had rehab; one group also learned “select Tai Chi moves” twice a wk. for 6 wks; instructor not identified	Function: ADLs (standard self-care scale) Adverse Events: Not reported Drop Outs: All completed the trial
Xu 2018 [101]	Hospital in mainland China (Chinese)	Single centre comparative trial (*n* = 98) Participants were on av. 38 yrs. old +/− 5 yrs. Severity of TBI not reported from MVA and other accidents. Time from TBI not reported (assumed ambulatory)	Add-on design: both grps had rehab; one group also learned Tai Chi. A nurse taught Tai Chi at least five times per wk. for 3 mos.	Function: FMA, Barthel Index for ADLs Adverse Events: No adverse events reported Drop-outs: Not reported
Hwang 2019 [100]	Home intervention in Taiwan (Chinese)	Single centre RCT (*n* = 96) Participants were 55 yrs. and older, post-discharge from hospital with TBI associated with mild/mod-severe cognitive impairment (assumed ambulatory)	An instructor with at least 10 years experience taught the 8-Form Yang style Tai Chi for 50 min 1/wk. and encouraged self-practice 3/wk. for 6 mo with a 6 mo follow-up. Two control groups: usual care and computerized cognitive training	Function: Hand-grip strength, Sit to stand, balance, ADLs, GOSE Cognitive: MDSR, MMSE, TICS-M, TMT A&B, CEDS Adverse Events: Not reported Drop Outs: 74% completed the two follow-up visits in this one-year study

*ADLs* Activities of daily living, *Av* Average, *CEDS* Centre for Epidemiologic Depression Scale, *FMA* Fugl-Meyer Assessment, *GOSE* Glasgow Outcome scale, extended, *grp* Group, *MMSE* Mini Mental Status Examination, *MOS-SF-36* General physical, social and mental well-being, *MDSR* Mattis Dementia Rating Scale, *min* Minutes, *mod* Moderate, *Q* Questionnaire, *RCT* Randomized controlled trial, *RSES* Rosenberg self-esteem scale, *TICS-M* Modified Telephone Interview of Cognitive Status, *TMT A&B* trail-making test, types A and B, *TBI* traumatic brain injury, *UK* United Kingdom, *VAMS* Visual analogue mood scale, *wk* Week, *Yrs* years

A meta-analysis was not performed given the limited number of trials, as well as the heterogenicity in population characteristics, intervention types, duration of intervention, and outcome measures.

### Risk of bias and GRADE assessment of randomized and non-randomized controlled trials

The RoB 2.0 tool was used to assess risk of bias in the three RCTs (Table 4). Given the nature of Tai Chi, research participants were unable to be blinded to the study intervention; however, the person assessing the outcome measures were blinded to group allocation. Two RCTs had full scores and were considered to have a low risk of bias [98, 100]. There was some risk of bias in one RCT due to the method used for randomization [102].

**Table 4:** Risk of Bias and GRADE assessment of randomized controlled trials of Tai Chi and Qigong for traumatic brain injury

Study	RoB2						GRADE
	Randomization process	Deviations from the intended interventions	Missing outcome data	Measurement of the outcome	Selection of the reported result	Overall	Certainty of evidence
Hwang 2019 [100]	Low risk	Low risk	Low risk	Low risk	Low risk	Low risk	High
Blake 2009 [98]	Low risk	Low risk	Low risk	Low risk	Low risk	Low risk	Moderate
Gemmell 2006 [102]	Some concerns	Low risk	Low risk	Low risk	Low risk	Some concerns	Moderate

The ROBINS-I tool was used to assess risk of bias in the two non-RCTs (Table 5). One trial had a moderate risk of bias as it did not consider confounding variables, and there were moderate concerns regarding selective reporting of outcome measures [101]. The other trial was considered to have a serious risk of bias as it also did not report on confounding variables and there were moderate concerns regarding the measurement of outcomes and the selection of reported results [99].

**Table 5:** Risk of bias and GRADE assessment of non-Randomized controlled trials of Tai Chi and Qigong for traumatic brain injury

Study	ROBINS-I								GRADE
	Confounding factors	Selection of participants	Measurement of interventions	Departure from intended interventions	Missing data	Measurement of outcomes	Selection of report results	Overall	Certainty of evidence
Xu 2018 [101]	Moderate risk	Low risk	Low risk	Low risk	No info	Moderate risk	Moderate risk	Moderate risk	Low
Manko 2013 [99]	Serious risk	Low risk	Low risk	Low risk	Low risk	Moderate risk	Moderate risk	Serious risk	Very low

The GRADE tool was used to assess all five trials (Table 4, Table 5). The certainty of evidence generated by the three RCTs was higher than the two non-RCTs as the three RCTs had lower risk of biases. One RCT met all domain requirements and was assessed to have a high certainty of evidence [100]. The other two RCTs’ quality of evidence was determined to be of moderate quality due to concerns with imprecision [98, 102]. Risk of bias and publication bias, with the addition of imprecision for one non-RCT [99], were the reasons for the lower scores for the non-RCTs [101].

Sub-group analyses and a funnel plot were not conducted due to insufficient data.

## Primary outcome measures

The most common primary outcome measures were functional status (activities of daily living and physical functioning), followed by psychological indicators and then cognitive function. “Return to normal activities” was not measured in any of the trials, nor were vestibular symptoms.

### Functional status

Xu assessed physical functioning and disability in 98 patients with TBI after a 3-month intervention of Tai Chi plus usual care or usual care alone. The Fugl-Meyer Assessment (FMA) was used to assess physical functioning, proprioception, balance, and joint pain, while the Barthel Index was used to assess disability. Statistically significant differences were identified between the Tai Chi and control groups (*p* = < 0.05) for both the FMA and Barthel Index scores [101].

Gemmell and Leathem used the SF-36 to assess physical function in 18 people with TBI after a 6-weeks of Tai Chi or being on a waiting list. Results showed no significant difference between the exercise and control group, except the Tai Chi group reported less difficulty performing usual activities due to less emotional turmoil than the control group [102].

Using the Standard Self-Care Scale to assess ADLs, Manko and colleagues evaluated the effectiveness of Tai Chi plus usual care vs usual care alone for 40 patients aroused from a prolonged coma after a severe TBI over a 6-week period. Within the Tai Chi group, there was improvement in reported self-care capacities, but the results did not reach statistical significance (*p* = 0.054) [99].

### Psychological status

Gemmell and Leathem used the Rosenberg self-esteem scale (RSES) and the visual analogue mood scale (VAMS) to assess self-esteem and mood as primary outcomes. No significant between group differences were found on the RSES [102]. Significant improvements within the Tai Chi group were reported for the VAMS dimensions of fear, confusion, sadness, anger, energy, happiness, and tension, but no significant difference was found for tiredness. Data from the waitlist control group was not reported [102].

Blake and Batson used the 12-item General Health Questionnaire to assess 20 people with TBI who participated in an 8-week intervention of Qigong vs social and leisure activities. The exercise group had significantly lower mood scores at the follow up period (*p* = 0.042), which indicated better mood [98].

### Cognitive status

Hwang and colleagues compared cognitive function post-TBI as a primary outcome measure in 96 people aged 55 years and older in 1 of 3 intervention groups: usual care (education promoting physical and mental activities), Tai Chi, or a computerized cognitive training (CCT) program. There was a 6-month intervention period and a follow-up 6 months later. Cognitive function was compared within each group using the Mattis Dementia Rating Scale (MDRS), Mini-mental status examination (MMSE), Modified Telephone Interview of Cognitive Status (TICS-M), and the trail-making test A and B (TMT). While there were no significant changes in any cognitive outcome in the usual care group, there was a significant improvement in the MDRS and MMSE at the 6 months in the CCT and Tai Chi groups, as well as improvement in the TMT B in the CCT group. There was no improvement in the TICS-M in either the Tai Chi or CCT group. At 1 year, the only significant sustained change was in the MDRS score in the Tai Chi group [100].

## Secondary outcome measures

The most common secondary outcomes measures documented in these studies were functional and psychological status.

Hwang and colleagues investigated ADLs, physical function (by handgrip strength and 5 sit-to-stands), disability using the Extended Glasgow Outcome Scale (GOSE), and depressive symptoms using the Center for Epidemiology Studies Depression Scale. Their results indicated that all groups significantly improved their GOSE scores by 12 months. The Tai Chi group had reduced time to complete 5 sit-to-stands at 6-months [100] and the CCT group improved hand grip strength, but there were no significant difference in all three groups for ADLs or depressive symptoms [100].

Through the Physical Self-Description Questionnaire, Blake and Batson measured physical functioning and self-esteem, as well as measured social support with the Social Support for Exercise Habits Scale. Their results indicated there was no significant difference between the two groups in physical functioning, but there was a significant improvement in self-esteem within the Tai Chi group (*p* = 0.017) [98]. There was no difference between Tai Chi versus controls for the level of social support [98].

Using the SF-36 to measure mental and emotional health, Xu reported statistically significant (*p* = 0.05) higher scores in the Tai Chi group [101]. Two trials [99, 102] did not investigate any secondary outcome measures.

## Adverse events

One RCT [98] reported that no adverse events occurred in either the intervention or control group. The other four trials did not report on adverse events.

## Discussion

To our knowledge, this is the first systematic review that has assessed the effectiveness of Tai Chi and Qigong for the treatment of TBI. We identified five trials from around the world, three RCTs and two non-RCTs. Overall, the four Tai Chi trials showed improved functional, psychological, and cognitive status in TBI patients. One RCT had a low risk of bias and a high degree of certainty and the other had some concerns about bias and a moderate degree of certainty. Not unexpectedly, the two non-RCTs had moderate to high risk of bias, and lower certainty of evidence scores. The one trial on Qigong, showed improved psychological function. Although it had a low risk of bias and a moderate degree of certainty, it was a small study and confirmatory evidence is indicated.

Several limitations merit consideration. The current standard of care for TBI is a gradual return to play, school, work, or usual activities [14, 15]. None of the five Tai Chi/Qigong trials measured return to usual activities. However, since all the trials were published before 2020, it is not surprising they did not reflect this recent patient-oriented outcome. Two non-RCTs investigated physical function and ADL function and found Tai Chi had a beneficial effect [99–101]; but due to non-randomization, their results should be interpreted with caution. High quality RCT evidence is indicated to assess Tai Chi/ Qigong for return to usual activities after TBI. The GOSE and Disability Rating Scale (DRS) are widely used measures of global functional status in TBI research, but it is acknowledged that they may lack granularity [18]. Likewise, it would be useful to assess vestibular function and inflammatory markers in future trials.

The trials covered different severities of TBI in a single study, which may mask the lack of efficacy for patients with a certain level of TBI severity. The trials included a large range in time from TBI before the intervention began, ranging from post-discharge from hospital to 40 years later. This discrepancy makes it difficult to determine the best time to initiate intervention post-TBI. Furthermore, the trials covered different styles, frequency, and duration of Tai Chi and Qigong interventions. These variations make it difficult to determine if one type of Tai Chi or Qigong is better than others, as well as determining the optimal length of intervention. Although some trials included young, middle-aged, and older adults, most were older adults. Since a growing proportion of patients with TBI are children and adolescents with sport-related injuries [3, 4, 6, 7]; the use of Tai Chi in this younger population remains unknown. Finally, only one trial reported on adverse events and found that none were experienced by either the exercise or control group [98]. Tai Chi is considered to be a safe practice [73]. Since Qigong is based on similar principles it is likely safe as well, although a systematic review assessing this is still pending [74]. In future studies of both Tai Chi and Qigong, adverse events should be carefully documented.

There are several additional recommendations that could be made to strengthen future studies. Future trials should include careful documentation of how the diagnosis of TBI was made. It would be useful to focus on a specific severity of TBI and a specific time from injury to initiation of intervention to allow for greater precision in the generalizability of results. There is no evidence yet to indicate when Tai Chi is best introduced into a TBI treatment plan. Future studies should determine whether Tai Chi is best introduced early, before complications become chronic, or later, when Tai Chi can help maintain the benefits of vestibular rehabilitation once active treatment is complete. It is a now a best practice to explicitly state and standardize the description of the Tai Chi intervention (e.g., form, style, frequency and qualifications of the instructor) [103]. Six-week interventions may be too short; longer trials and longer follow-up periods are indicated, especially for those with moderate TBI or who have persistent symptoms. Furthermore, studies should reflect the latest advances in TBI management, so in addition to return to normal activities, including vestibular status and pre and post assessment of inflammatory markers are also indicated.

In conclusion, TBI often has significant physical, psychological, and cognitive impacts on patients. Current treatments, while beneficial, are not entirely effective as the majority of patients continue to experience some symptoms one-year post-injury [18]. Tai Chi/Qigong are known for improving general well-being and mitigating the effects of chronic disease. The use of Tai Chi/Qigong for injury, such as TBI, is a new frontier. Considering the high level of certainty from one RCT and the beneficial effects found in all four trials on Tai Chi, there is a sufficient signal to merit conducting a multi-centre trial on Tai Chi for TBI to test Tai Chi against current trends in the management of TBI. Another single centre RCT is indicated to confirm the initial findings for Qigong.

### Supplementary Information

**Additional file 1.**

## Data Availability

Appendix 1 includes the Chinese and English database search strategy. Otherwise, the datasets used and/or analysed during the current study are available from the corresponding author on reasonable request.

## Abbreviations

ADLs: Activities of daily living
CEDS: Centre for epidemiologic depression scale
CCT: Computerized cognitive training
CT: Computer tomography
DRS: Disability rating scale
FMA: Fugl-meyer assessment
GOSE: Extended glasgow outcome scale
GRADE: Grading of recommendation, assessment, development, and evaluation
MDRS: Mattis dementia rating scale
MMSE: Mini-mental state examination
MOS-SF-36: Medical outcomes scale short form
MVA: Motor vehicle accident
Non-RCTs: Non-randomized controlled trials
PRISMA: Preferred reporting items for systematic reviews and meta-analysis
RCT: Randomized controlled trial
RoB 2.0: Risk of bias tool
ROBINS-I: Non-randomized studies-of interventions
RSES: Rosenberg self-esteem scale
TBI: Traumatic brain injury
TICS-M: modified Telephone Interview of Cognitive Status
TMT: Trail making test
VAMS: Visual analogue mood scale
