# Supplementary material for: Are Tai Chi and Qigong effective in the treatment of traumatic brain injury? A systematic review
Source: BMC Complement Med Ther. 2024 Feb 6;24:78. doi: 10.1186/s12906-024-04350-3 (PMC10845721; doi:10.1186/s12906-024-04350-3)
Supplement: Supplementary file 1 — Additional file 1. [file 12906_2024_4350_MOESM1_ESM.docx]

**Appendix 1: English Database Search Strategy Results as of January 25, 2023**

| Ovid MEDLINE | | |
| --- | --- | --- |
| 1 | exp Craniocerebral Trauma/ | 175995 |
| 2 | ((head or crani* or capitis or brain* or forebrain* or skull* or hemisphere or intracran* or orbit* or cerebr*) adj2 (injur* or trauma* or lesion* or damage* or wound* or destruction* or oedema* or edema* or fracture* or contusio* or pressur*)).ti,ab,kf. | 231687 |
| 3 | (mtbi or tbis or tbi).ti,ab,kf. | 33230 |
| 4 | concuss*.ti,ab,kf. | 12472 |
| 5 | commotio*.ti,ab,kf. | 677 |
| 6 | or/1-5 | 330001 |
| 7 | Tai Ji/ or Qigong/ | 1637 |
| 8 | (t'ai chi* or tai chi* or tai ji* or taiji* or qi gong* or qigong* or chi kung* or chikung*).ti,ab,kf. | 3270 |
| 9 | or/7-8 | 3386 |
| 10 | 6 and 9 | 16 |

| CINAHL | | |
| --- | --- | --- |
| 1 | (MH "Head Injuries+") | 47804 |
| 2 | TI ( ((head or crani* or capitis or brain* or forebrain* or skull* or hemisphere or intracran* or orbit* or cerebr*) N2 (injur* or trauma* or lesion* or damage* or wound* or destruction* or oedema* or edema* or fracture* or contusion* or pressur*)) ) OR AB ( ((head or crani* or capitis or brain* or forebrain* or skull* or hemisphere or intracran* or orbit* or cerebr*) N2 (injur* or trauma* or lesion* or damage* or wound* or destruction* or oedema* or edema* or fracture* or contusion* or pressur*)) ) | 60148 |
| 3 | TI ( (mtbi or tbis or tbi) ) OR AB ( (mtbi or tbis or tbi) ) | 11224 |
| 4 | TI concuss* OR AB concuss* | 6620 |
| 5 | TI commotio* OR AB commotio* | 210 |
| 6 | S1 OR S2 OR S3 OR S4 OR S5 | 81808 |
| 7 | (MH "Tai Chi") | 2404 |
| 8 | (MH "Qigong") | 818 |
| 9 | TI ( (t’ai chi* or tai chi* or tai ji*or taiji* or or qi gong* or qigong* or chi kung* or chikung*) ) OR AB ( (t’ai chi* or tai chi* or tai ji*or taiji* or or qi gong* or qigong* or chi kung* or chikung*) ) | 3059 |
| 10 | S7 OR S8 OR S9 | 3982 |
| 11 | (S7 OR S8 OR S9) AND (S6 AND S10) | 29 |

| EMBASE | | |
| --- | --- | --- |
| 1 | exp head injury/ | 330959 |
| 2 | ((head or crani* or capitis or brain* or forebrain* or skull* or hemisphere or intracran* or orbit* or cerebr*) adj2 (injur* or trauma* or lesion* or damage* or wound* or destruction* or oedema* or edema* or fracture* or contusion* or pressur*)).ti,ab,kw. | 301995 |
| 3 | (mtbi or tbis or tbi).ti,ab,kw. | 54883 |
| 4 | Concuss*.ti,ab,kw. | 16983 |
| 5 | Commotio*.ti,ab,kw. | 755 |
| 6 | 1 or 2 or 3 or 4 or 5 | 476913 |
| 7 | exp Tai Chi/ | 3816 |
| 8 | exp Qigong/ | 1096 |
| 9 | (t'ai chi* or tai chi* or tai ji*or taiji* or qi gong* or qigong* or chi kung* or chikung*).ti,ab,kw. | 4251 |
| 10 | 7 or 8 or 9 | 5449 |
| 11 | 6 and 10 | 46 |

| OVID Cochrane Library | | |
| --- | --- | --- |
| 1 | exp Craniocerebral Trauma/ | 4231 |
| 2 | ((head or crani* or capitis or brain* or forebrain* or skull* or hemisphere or intracran* or orbit* or cerebr*) adj2 (injur* or trauma* or lesion* or damage* or wound* or destruction* or oedema* or edema* or fracture* or contusion* or pressur*)).ti,ab. | 13570 |
| 3 | (mtbi or tbis or tbi).ti,ab. | 3223 |
| 4 | Concuss*.ti,ab. | 877 |
| 5 | Commotio*.ti,ab. | 14 |
| 6 | 1 or 2 or 3 or 4 or 5 | 16018 |
| 7 | exp Tai Ji/ | 411 |
| 8 | exp Qigong/ | 99 |
| 9 | (tai chi* or tai ji*or taiji* or qi gong* or qigong* or chi kung* or chikung*).ti,ab. | 2092 |
| 10 | 7 or 8 or 9 | 2126 |
| 11 | 6 and 10 | 14 |
